# Ultra‐Processed Food Policies and Global Nutrition Equity: The Case for a Feasibility‐Grounded, Differentiated Governance Supplement to Nova

**DOI:** 10.1111/nbu.70061

**Published:** 2026-07-07

**Authors:** Jimmy Chun Yu Louie

**Affiliations:** ^1^ Discipline of Dietetics, School of Health Sciences Swinburne University of Technology Hawthorn Victoria Australia

**Keywords:** dietary governance, food systems, global health, humanitarian nutrition, mandatory reformulation, micronutrient fortification, Nova classification, nutrition equity, ultra‐processed foods

## Abstract

The Nova food classification system has substantially advanced nutritional epidemiology by identifying industrial food processing as a meaningful determinant of dietary quality and health outcomes, independent of nutrient composition. Yet governance proposals derived from Nova face a structural tension inadequately resolved in the nutrition policy literature: for hundreds of millions of people across low‐ and middle‐income countries (LMICs), humanitarian settings, and food‐insecure urban environments, processed foods perform caloric, micronutrient, and therapeutic functions that are not substitutable within realistic policy timeframes. This analysis examines Nova Group 4's classification heterogeneity, the policy operability gaps it creates, and the food security realities that blanket ultra‐processed food (UPF) reduction recommendations systematically ignore. The argument is not against Nova's epidemiological insights but for supplementing Nova‐based guidance with a product‐differentiated, feasibility‐grounded governance framework—one that deploys mandatory reformulation, evidence‐based fiscal and marketing instruments, protected fortification infrastructure, and food system investment as the instruments through which public health ambition becomes practically achievable.

## 1 Introduction

The imperative to improve dietary quality for the world's 8 billion people sits at the intersection of two evidence bases that have developed largely in parallel but rarely in productive dialogue. The nutritional epidemiology of ultra‐processed foods (UPFs), anchored in the Nova classification system developed by Monteiro et al. (2018, 2010), has generated compelling evidence that the degree and nature of industrial food processing represent a meaningful and independent determinant of health outcomes. The food security literature, grounded in agricultural economics, development studies, and humanitarian nutrition, has documented with equal rigour the structural functions that processed and shelf‐stable foods perform in feeding populations for whom the dietary ideals of nutritional epidemiology are structurally inaccessible (Gallegos 2025).

The epidemiological evidence linking Nova‐classified UPFs to adverse health outcomes is now substantial. Prospective cohort studies across Europe, the Americas, Asia, and Australia have identified consistent associations between higher UPF consumption and increased risks of obesity, type 2 diabetes, cardiovascular disease, multiple cancers, depression, cognitive health, and all‐cause mortality (Lane et al. 2024; Rico‐Campà et al. 2019; Srour et al. 2020; Srour et al. 2019; Smith et al. 2026). Recent randomised controlled trials have now examined the effects of UPF consumption (Dicken et al. 2025; Hall et al. 2019; Hamano et al. 2024; Preston et al. 2025), with most of these finding that *ad libitum* consumption of ultra‐processed diets resulted in significantly higher energy intake and greater weight gain than matched unprocessed food diets, even when macronutrient composition was controlled.

The 2025 Lancet Series on UPFs and Human Health, authored by 43 international experts, synthesises this evidence and proposes governance frameworks spanning mandatory reformulation, front‐of‐pack labelling, marketing restrictions, fiscal instruments, corporate power constraints, and agricultural subsidy redirection (Baker et al. 2020; Monteiro et al. 2025; Scrinis et al. 2025).

Yet this governance ambition confronts a structural reality that the Lancet Series and cognate governance proposals have acknowledged in principle but not resolved in practice. For many populations the structural roles of processed foods extend well beyond discretionary consumption. This analysis operates across three analytically distinct levels: Nova as an epidemiological exposure measure (where its validity is strongest); as a food classification instrument (where heterogeneity constrains product‐level resolution); and as a policy enforcement tool (where operability gaps are most acute). The critique applies differentially, and is sharpest at the second and third levels.

The argument advanced is not that Nova's epidemiological insights should be dismissed—they should not—but that those insights require supplementation with a product‐differentiated, feasibility‐grounded governance framework that can accommodate the full diversity of nutritional roles performed by processed foods across the global food system.

## 2 The Nova Framework: Contributions and Limitations

Nova's most significant contribution to nutritional science was the proposition that the degree and nature of industrial food processing represent a distinct and nutritionally meaningful dimension of food quality, independent of nutrient composition (Monteiro et al. 2018). This reorientation had genuine analytical power: it helped explain why nutrient‐focused interventions had repeatedly failed to reverse obesity and non‐communicable diseases (NCDs) trajectories (Scrinis 2020; Lawrence 2023), and it provided a framework for examining the political economy of food processing, a framing validated by subsequent evidence of systematic corporate lobbying and research interference (Baker et al. 2025).

Despite its conceptual contributions, Nova has been subject to persistent and well‐founded criticism regarding the consistency, reproducibility, and operational stability of its Group 4 classifications (Gibney et al. 2017; Louie 2025; Ludwig 2025; Marino et al. 2021; Robinson and Johnstone 2024; Louie 2026c). The boundary between Group 3 (processed foods) and Group 4 (UPFs) is neither empirically precise nor consistently applied across studies (Dickie et al. 2022): the same sourdough bread may be classified Group 3 or Group 4 depending on the analytical team, the ingredient list format, and the Nova version applied (Kahleova et al. 2026; Louie 2025).

More consequentially, Group 4 encompasses products with radically different nutritional profiles, health implications, and food system functions. At one extreme sit sugar‐sweetened beverages (SSBs), products with no essential nutritional role and strong evidence of harm across consumption levels (Yan et al. 2022; Malik et al. 2010). At the other sit ready‐to‐use therapeutic foods (RUTFs) designed to treat severe acute malnutrition in children under five in humanitarian settings, and fortified complementary foods for infants that address iron, zinc, and vitamin A deficiency in populations where dietary diversity is structurally constrained (Collins et al. 2006; UNICEF 2023). Classifying these products identically is not a methodological oversight; it is a fundamental analytical limitation that renders Nova Group 4, as a regulatory category, both scientifically heterogeneous and operationally challenging.

A common response to this heterogeneity critique is that the relevant harm derives from the overall UPF dietary pattern rather than any individual product (Monteiro et al. 2025). This is a coherent epidemiological argument, and the pattern‐level evidence is genuinely persuasive. The difficulty is that governance must ultimately operate on individual products: regulatory instruments—taxes, warning labels, marketing restrictions, school food bans, reformulation mandates—apply to specific products, not to dietary patterns (Mozaffarian et al. 2018; Popkin et al. 2024). If the regulatory unit is necessarily the individual product, the heterogeneity of Group 4 is not an unhelpful distraction but a central regulatory challenge that pattern‐level arguments cannot resolve.

The epidemiological evidence underpinning Nova‐informed governance, while extensive, carries methodological limitations that are insufficiently foregrounded in advocacy communications (Louie 2025; Louie 2026b). Virtually all prospective cohort evidence is observational and subject to confounding by socioeconomic status, lifestyle, and dietary quality factors that co‐vary with UPF consumption (Gibney et al. 2017; Louie 2025; Ludwig 2026). Whether ultra‐processing per se*,* distinct from the nutritional characteristics it typically represents, independently predicts outcomes remains unclear (Dickie et al. 2022; Pagliai et al. 2021). These limitations are shared by much of nutritional epidemiology and do not invalidate Nova's contributions; they do, however, argue for epistemic humility in translating association evidence into prescriptive governance frameworks, particularly when those frameworks would restrict foods serving essential food security functions.

## 3 Structural Food Security Realities and the Governance Gap

### 3.1 The Scale of Structural Dependency

Any governance framework aspiring to global applicability must begin with the scale of food insecurity it would operate within (Willett et al. 2019). As of 2023, approximately 733 million people face acute food insecurity, and over 2 billion experience moderate to severe food insecurity (FAO et al. 2023). Urban populations in LMICs will constitute the majority of net urban growth, predominantly in food environments structurally dominated by affordable processed foods (UNDESA 2018).

**FIGURE 1:**
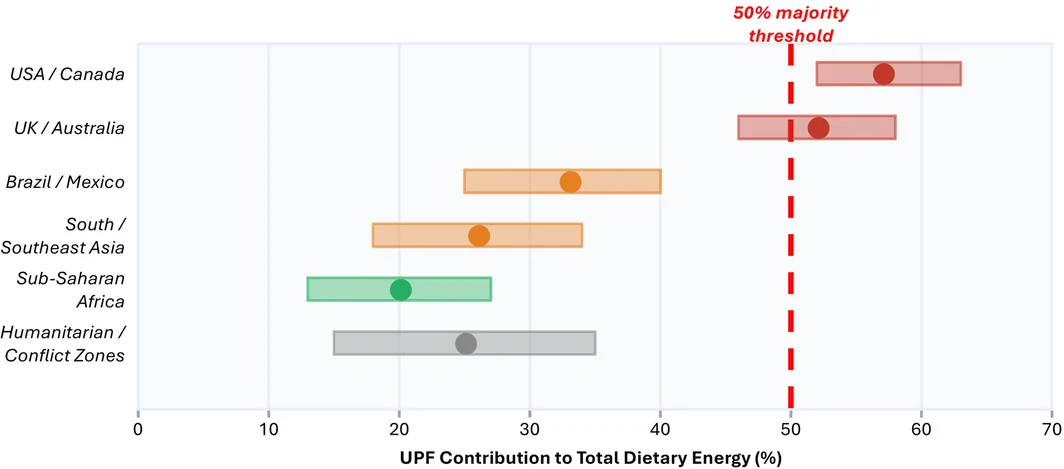
UPF contribution to total dietary energy by world region. Shaded bars = estimated range, circles = midpoint. The 50% threshold (dashed red) marks the point at which UPFs constitute the majority of caloric intake.

UPFs supply 55%–60% of dietary energy in the United States and Canada, 50%–57% in the United Kingdom and Australia and 30%–40% in Brazil and Mexico, proportions rising across virtually every country with available trend data (Popkin and Reardon 2018; Monteiro et al. 2013; Machado et al. 2020) (Figure 1). These figures establish not only the public health challenge but also the scale of structural dependency any transition must navigate. For lower‐income households, whole‐food diets meeting nutritional standards cost 1.5–2.5 times more per calorie than UPF‐dominated diets in high‐income countries (Jones et al. 2014; Headey and Alderman 2019). Governance recommendations calling for agricultural subsidy reform and local food system investment (Baker et al. 2025; Scrinis et al. 2025) are correct in principle but are decade‐scale structural interventions that provide no solution to immediate food security constraints.

### 3.2 Cold‐Chain Infrastructure and Shelf‐Stability Dependency

A core implicit assumption of Nova‐informed dietary recommendations is that fresh, minimally processed whole foods represent a practically accessible alternative (Monteiro et al. 2015). This assumption fails across large portions of the global food system. Adequate cold‐chain infrastructure remains absent or severely inadequate across much of sub‐Saharan Africa, South Asia, and parts of Southeast Asia and Latin America (Makule et al. 2022; Amjad et al. 2023). Approximately 40% of food produced in developing countries is estimated to be lost post‐harvest before reaching consumers, rising above 50% for perishable horticultural products in sub‐Saharan Africa (FAO 2011; World Bank 2011). In the absence of reliable cold‐chain infrastructure, shelf‐stable processed foods are not dietary compromises; they are nutritional necessities (Reardon et al. 2021; Claro et al. 2010).

### 3.3 The Micronutrient Delivery Architecture

An estimated 2 billion people are affected by micronutrient deficiencies; iron, iodine, vitamin A, folate, and zinc generating the largest preventable morbidity and mortality burdens (Stevens et al. 2013; World Health Organization 2009). The primary mechanism addressing these deficiencies at population scale in most of the world is mandatory micronutrient fortification of staple food vehicles (Ashraf 2025; Demekas and Rigutto‐Farebrother 2024; Olson et al. 2021). Iodised salt programmes have dramatically reduced iodine deficiency disorders over three decades; iron and folate fortification of wheat flour has contributed to reductions in anaemia and neural tube defects; vitamin A fortification of oils has reduced child mortality where deficiency remains prevalent (Keats et al. 2021).

It is essential to note the correct Nova classification of these vehicles. Under the 2016+ Nova definition (Monteiro et al. 2024), iodised salt, fortified oils, and fortified flour used as bulk culinary staples are classified as Nova Group 2 (processed culinary ingredients with added vitamins or minerals)—not Group 4. The governance risk does not arise from direct product‐level targeting of these Group 2 vehicles, but from consumer‐facing UPF reduction messaging that does not faithfully reproduce the Group 2/4 distinction—creating collateral communication risk in populations dependent on these vehicles for micronutrient adequacy. The British Nutrition Foundation has similarly emphasised that any UPF governance framework must avoid inadvertently undermining fortification programmes on which nutritionally vulnerable populations depend (British Nutrition Foundation 2024).

Packaged fortified products that are genuinely Group 4—infant formula, fortified complementary foods, fortified breakfast cereals—do, however, play significant roles in micronutrient delivery, particularly among populations in high‐income countries and in transitional food environments. The Copenhagen Consensus (2012) ranked micronutrient fortification among the highest benefit–cost ratio interventions in global health (9:1–30:1). Any UPF governance framework that applies blanket restriction messaging to Group 4 without explicit carve‐outs for these products risks inadvertent harm to populations dependent on them.

### 3.4 Humanitarian Settings and the Life‐Saving UPF

Severe acute malnutrition (SAM), affecting an estimated 13.6 million children under five globally (UNICEF 2023), represents the world's most immediately life‐threatening form of malnutrition. The primary treatment modality for uncomplicated SAM in community settings—endorsed by WHO, UNICEF, WFP and FAO—is ready‐to‐use therapeutic food (RUTF), most commonly a lipid‐based nutrient supplement containing peanut paste, milk powder, sugar, vegetable oil and micronutrients (World Health Organization 2013). RUTF saves children's lives at approximately USD 45–60 per child treated, with cure rates exceeding 90% in well‐functioning programmes.

RUTF is correctly classified as Nova Group 4 under the industrial formulation criterion, this is not a misclassification. The governance failure is not that RUTF belongs in the wrong category, but that current Nova‐aligned governance proposals apply identical reduction messaging to RUTF and sugar‐sweetened beverages despite their diametrically opposed nutritional functions and humanitarian necessity. The word ‘therapeutic’ does not appear in any current major Nova‐aligned governance proposal, and no explicit functional carve‐out for humanitarian nutrition products appears in the policy frameworks that have followed the Nova evidence synthesis. For a governance framework aspiring to global application, this is not a peripheral omission, it is a foundational gap.

Infant formula, oral rehydration solutions, clinical nutrition products for patients with specific metabolic requirements, and texture‐modified meal replacements for people with dysphagia are all classified as Nova Group 4 UPFs, despite being essential to preventing morbidity and mortality in specific populations (Cichero et al. 2017; Gibney et al. 2017; Nalin 2021). The elimination of these products would predictably worsen health outcomes, underscoring a fundamental limitation of processing‐based classification systems (Gibney et al. 2017; Jones 2019). A regulatory carve‐out adequate for humanitarian nutrition products would require, at minimum: a positive list approach identifying WHO‐endorsed therapeutic nutrition formulations as explicitly exempt from Group 4 reduction guidance; mandatory exemption clauses in national dietary guidelines adopting Nova‐based recommendations; and explicit language in front‐of‐pack labelling regulations excluding therapeutic nutrition products from warning label requirements. Maintenance of such a list would appropriately fall under existing WHO/UNICEF/WFP technical committee structures.

### 3.5 Time Poverty, Energy Poverty and the Convenience Imperative

Nova‐informed recommendations frequently invoke home cooking from minimally processed ingredients as the practical alternative to UPF consumption (Brazilian Ministry of Health 2014; Monteiro et al. 2025). Time available for food preparation competes with paid employment, caregiving responsibilities and transportation in households where these burdens fall disproportionately on women (Monsivais et al. 2014; Daniel 2016; Rao et al. 2026). Studies consistently find that time available for home cooking is negatively associated with household income, hours worked and housing insecurity—precisely the characteristics that also predict higher UPF consumption and poorer dietary quality (Monsivais et al. 2014).

In LMIC contexts, the time poverty dimension is compounded by energy poverty: in households without reliable access to cooking fuel, affecting approximately 2.4 billion people globally (IEA 2020), the time and fuel cost of preparing minimally processed whole foods represents a genuine barrier that shelf‐stable processed foods circumvent.

This constraint is not confined to low‐income countries. In England, approximately 13% of households are classified as fuel poor, with Black and minority ethnic households disproportionately represented (Department for Energy Security and Net Zero (UK) 2024). The UK Food Foundation has reported that more than half of UK households were worried that higher energy prices would leave them with less money to buy food (The Food Foundation 2021). In parallel, housing insecurity introduces additional structural constraints on food practices. In England, the number of children living in temporary accommodation reached over 151 000 in 2024, reflecting a rapidly growing population exposed to unstable and often unsuitable housing conditions (Shelter 2024). Such circumstances frequently involve limited or shared kitchen facilities, overcrowding, and restricted storage, all of which constrain the feasibility of routine food preparation. In the United States, 13.7% of households were food insecure in 2024, with food‐energy trade‐offs documented across low‐income urban populations (Rabbitt et al. 2025). Within this context, trade‐offs between essential expenditures, including food, energy, and housing, are widely documented features of food insecurity, particularly among low‐income households.

These dynamics illustrate a broader point: the assumption that home cooking from minimally processed ingredients is a readily available alternative to UPF consumption does not hold uniformly, even in high‐income settings. Structural constraints, ranging from fuel poverty and income volatility to inadequate housing infrastructure, can limit both the capacity to purchase raw ingredients and the ability to prepare them. At a global level, the role of infrastructure in shaping dietary behaviour is well established.

While the specific mechanisms differ, the underlying principle, that material constraints on cooking capacity shape dietary patterns, applies across contexts (IEA 2020). The assumption that home cooking from minimally processed ingredients is a readily available alternative to UPF consumption fails for fuel‐poor and food‐insecure households in high‐income countries for precisely the same structural reasons it fails across LMIC contexts—an equivalence that UPF governance proposals calibrated to population dietary averages systematically obscure.

### 3.6 The Absence of a Credible Transition Strategy

Recent governance proposals invoke the language of a ‘just transition’ to low‐UPF diets (Baker et al. 2025; Scrinis et al. 2025), borrowed from fossil fuel phase‐out frameworks. In energy transition contexts, however, ‘just transition’ frameworks are accompanied by detailed sector‐by‐sector plans, costed support programmes for affected workers, defined timelines, and institutional accountability mechanisms. No comparable operational framework exists for UPF dietary transition. The question is not whether global diets should rely less on industrial ultra‐processing—they clearly should—but whether any realistic version of global food systems can deliver such a transition for 8 billion people within timeframes relevant to current health burdens, while protecting the most vulnerable. Current proposals do not demonstrate this and acknowledging that gap honestly is the prerequisite for developing governance frameworks that can. It should equally be acknowledged that the alternative framework proposed in Section 5 is similarly lacking in formal cost estimates; costing both approaches is a priority for future work.

## 4 Reformulation, Fortification and Product‐Differentiated Governance

Given the structural necessity of processed foods in the current global food system, improving the nutritional quality of UPFs that populations actually consume becomes a public health imperative. This is not a concession to the food industry; it is the logical consequence of taking both the epidemiological evidence and the food security evidence seriously.

The elimination of partially hydrogenated oils from the global food supply through mandatory bans represents the most successful example of UPF reformulation as public health strategy: in Denmark, cardiovascular mortality fell measurably within years of the *trans* fat ban's implementation (Restrepo and Rieger 2016; Amico et al. 2021), and WHO has targeted global elimination of industrial *trans* fats by 2023 (Steele et al. 2024). Systematic sodium reduction across major product categories has been modelled to generate population‐level cardiovascular disease reductions comparable in magnitude to pharmacological interventions (Cobiac et al. 2010).

A critical distinction separates mandatory reformulation, implemented through binding legislative standards, from voluntary programmes driven by industry initiative. Voluntary sodium reduction in the United Kingdom achieved modest, inconsistent reductions over a decade, with significant variation across companies and persistent reliance on industry goodwill vulnerable to commercial pressures (He et al. 2014). By contrast, mandatory *trans* fat elimination achieved near‐complete supply‐side transformation within years of implementation (Restrepo and Rieger 2016). The pattern is consistent: voluntary agreements produce modest, variable, difficult‐to‐verify progress; mandatory standards produce reliable, measurable, sustained change (Hyseni et al. 2017).

Mandatory micronutrient fortification must be treated as a non‐negotiable component of any food governance framework. Regulatory frameworks must explicitly distinguish between mandatory fortification of staple foods as public health infrastructure—which should be strengthened and expanded—and discretionary Group 4 products carrying neither essential nutritional functions nor food security roles. This distinction does not feature explicitly in any current major Nova‐aligned governance proposal, representing a significant gap between the equity aspirations of those proposals and their operational implications. Compositional reformulation standards should be holistic, addressing nutrient profiles across multiple dimensions simultaneously rather than single‐nutrient targets, to avoid unintended compositional substitutions that may introduce new harms.

The foregoing analysis converges on a structural conclusion: Nova Group 4 is too heterogeneous a category to serve as the operational unit for governance. The category ranges from demonstrably harmful, nutritionally empty products to nutritionally critical and life‐saving formulations. The tobacco analogy—invoked to justify urgent comprehensive action (Baker et al. 2025; Gearhardt et al. 2026; Gearhardt and DiFeliceantonio 2023)—is instructive regarding corporate political strategy but misleading regarding product governance: tobacco has no nutritional value, no food security role, and a dose–response disease relationship at any consumption level (Louie 2026a). The UPF category is nutritionally heterogeneous; the appropriate policy implication is differentiated regulation, not categorical elimination (Louie 2026d) (Table 1).

**TABLE 1:** Nova group 4 classification inconsistencies: Products where assigned classification diverges from nutritional role and food security function.

Product	Nova group	Classification issue	Food system implication	Correct governance approach
*Artisan sourdough bread*	Group 3 (original); Group 4 in many national applications	Classification driven by ingredient list depth, not health relevance	Boundary instability creates inconsistent regulatory signals	Classify by function; not process category alone
*Flavoured yoghurt*	Group 3 or Group 4 (many commercial versions)	Identical health profiles may straddle boundary depending on flavouring method	Reformulation incentives may conflict with Nova classification signals	Nutrient‐based compositional standards
*Fortified infant cereal*	Group 4	Nutritionally critical product classified identically to confectionery	No exception for clinical nutrition purpose under Group 4 reduction messaging	Explicit functional exemption required
*Ready‐to‐use therapeutic food (RUTF)*	Group 4	Life‐saving emergency nutrition product treated as equivalent to soft drink	Uncorrected by recent major advocacy documents including Monteiro et al. (2025)	Mandatory exemption for WHO‐endorsed therapeutic nutrition products
*Plant‐based meat analogues*	Group 4 (most versions)	Lower environmental footprint; often better nutritional profile than processed meat	Nova classification may inadvertently discourage environmentally beneficial transitions	Lifecycle assessment alongside Nova classification

*Note:* (Dickie et al. 2023; Gibney et al. 2017; Louie 2025; Marino et al. 2021; Kahleova et al. 2026). Note distinction between RUTF (correctly Group 4 but requiring functional carve‐out) and iodised salt/fortified oils (correctly Group 2, not Group 4, under 2016+ Nova).

## 5 Toward a Feasibility‐Grounded Governance Framework

The critique of Nova as a stand‐alone primary governance framework does not negate the need for robust food supply governance, it demands a more operationally grounded architecture than categorical classification systems alone can provide. A feasibility‐grounded supplement to Nova‐based guidance should be guided by five principles: (1) differentiation over categorisation—governance calibrated to the nutritional profile, harm evidence, and food system function of specific product categories; (2) equity‐centred design—protecting and improving food security for populations most dependent on processed foods, not merely optimising dietary choices for those with resources to act on healthy eating guidance; (3) evidence‐based prioritisation—instruments selected and sequenced by evidence strength, not theoretical coherence with a classification framework; (4) supply‐side and demand‐side complementarity—simultaneously addressing demand drivers and supply conditions that make whole food alternatives genuinely accessible and affordable; and (5) protection of critical nutritional infrastructure—explicit, binding protective mechanisms for products serving micronutrient delivery or therapeutic nutrition functions.

This framework extends beyond existing product‐differentiated governance proposals in the literature by explicitly integrating food security equity considerations—particularly the protection of nutritionally critical processed foods serving LMIC populations—as non‐negotiable governance design requirements, and by applying this framework to the specific governance gap created by Nova's categorical architecture. Political economy safeguards are essential: mandatory conflict‐of‐interest disclosure for compositional standard‐setting committee membership; independent scientific review of proposed compositional thresholds by bodies without industry representation; requirements for public consultation documentation; and mandatory post‐implementation evaluation with pre‐specified outcome measures.

The evidence base for specific governance instruments has grown substantially and provides a foundation for regulatory action not dependent on Nova's categorical architecture. Among demand‐side instruments, mandatory front‐of‐pack warning labels modelled on Chile's experience are well‐evidenced and should be expanded globally (Taillie et al. 2021). SSB taxes with revenue hypothecated to whole‐food subsidies or food security programmes have a robust evidence base and should be widely implemented (Colchero et al. 2016; Teng et al. 2019), with enforceable revenue recycling mechanisms to prevent regressivity impacts on lower‐income households (Colchero et al. 2016; Colchero et al. 2017). Marketing restrictions targeting nutritionally poor UPFs to children are among the most robustly evidence‐based interventions available (Boyland and Whalen 2015; Corvalán et al. 2019). Equity considerations are acute: SSB taxes are regressive if revenue is not hypothecated to low‐income food support; marketing restrictions disproportionately benefit children in low‐income households most exposed to UPF marketing.

Among available governance instruments, mandatory reformulation represents the highest priority on grounds of evidence strength, population reach, and equity impact. Unlike individual‐level dietary recommendations, mandatory reformulation changes the nutritional composition of the food supply itself, improving the diet of all who consume the affected products regardless of dietary knowledge, income, or agency. Priority targets include: sodium reduction across processed meats, bread, ready meals, and sauces; sugar reduction in SSBs, confectionery, and dessert products; elimination of residual industrially produced *trans* fats; and saturated fat reduction where replacement with unsaturated fats is technically feasible (Cobiac et al. 2010; He et al. 2014; Federici et al. 2019). For LMIC contexts, mandatory reformulation requires a staged approach: technical standard‐setting as a first step (achievable even with limited enforcement capacity), followed by mandatory declaration requirements, and ultimately binding compositional limits as regulatory capacity develops. All demand‐side governance instruments must be paired with supply‐side food system infrastructure investment (High Level Panel of Experts on Food Security and Nutrition 2017)—cold‐chain development in LMICs, urban fresh food market access, and smallholder production incentives—as the structural preconditions for progressively reducing UPF dependence without imposing nutritional harm (Table 2).

**TABLE 2:** Evidence‐based UPF governance framework: policy levers, mechanisms, evidence strength, equity considerations and priority contexts.

Policy lever	Mechanism	Evidence strength	Equity consideration	Priority context
*Mandatory reformulation* (Downs et al. 2013; Gressier et al. 2025)	Legislated compositional limits: sodium, sugar, trans fat; nutrient density floors	Strong	Context‐neutral; applicable across income levels	All settings; HIC first
*Interpretive FOP labels* (Taillie et al. 2021; Silva et al. 2022)	Mandatory warning labels: (Chile octagonal model); Nutri‐Score; Health Star Rating	Strong	Lower literacy populations need symbol‐based systems	Universal
*Marketing restrictions* (Dillman Carpentier et al. 2023)	Restrict UPF advertising to children; school zones; media watersheds	Moderate–Strong	Children in low‐income households disproportionately exposed	All income settings
*Fiscal instruments* (Andreyeva et al. 2022)	SSB taxes; sugar/salt taxes; subsidies for whole foods and legumes	Strong for SSB	Regressive if not hypothecated to low‐income food support	All settings
*Public procurement* (Niebylski et al. 2014)	Mandate whole food provision in schools, hospitals, government institutions	Moderate	High impact in food‐insecure household settings	All income settings
*Food system infrastructure* (Liao et al. 2024)	Cold‐chain investment; urban fresh food markets; smallholder incentives	Context‐specific; long‐term horizon	Structurally enables reduction of UPF dependence over time	LMICs; urban food deserts

*Note:* Note the inclusion of mandatory fortification protection — absent from most current governance frameworks—and explicit equity considerations for fiscal measures.

Abbreviations: FOP, front‐of‐pack; HIC, high‐income countries; LMICs, low‐ and middle‐income countries; SSB, sugar‐sweetened beverages.

## 6 Discussion

The critique of Nova's operational limitations must not be read as an argument for policy inaction. The evidence for harm from SSBs justifies fiscal and marketing interventions in virtually any policy context. The evidence for harm from high‐sodium processed diets justifies mandatory sodium reformulation targets. None of this requires Nova as a classification framework, it requires specific, evidence‐based regulatory instruments directed at specific product categories with specific evidence of harm.

The risk of the current Nova‐centric discourse is not merely that some recommendations are impractical in some contexts, it is that recognised impracticality generates political space in which all UPF governance is dismissed as infeasible or idealistic. The appropriate response is to advance governance instruments that are simultaneously evidence‐based, operationally grounded, and robust to the critique that they ignore the structural realities of global food security. A framework protecting mandatory fortification programmes, exempting therapeutic nutrition products, deploying mandatory reformulation as its primary near‐term instrument, and pairing demand‐side fiscal and labelling measures with supply‐side infrastructure investment is not a compromise of public health ambition—it is the realisation of that ambition in practically achievable form.

The path forward requires three conceptual reorientations. First, differentiation: replacing Nova's binary UPF/non‐UPF governance signal with a product‐differentiated logic calibrating regulatory ambition to harm evidence, food system function, and equity implications in specific population contexts. Second, proportionality: applying governance instruments at the scale and intensity justified by evidence, prioritising product categories with the strongest harm evidence and greatest population exposure, rather than pursuing comprehensive elimination of a heterogeneous category. Third, systems thinking: recognising that effective governance requires simultaneous action on supply‐side and demand‐side food system determinants, and that long‐term structural dietary improvement requires building food systems in which healthy, minimally processed diets are genuinely accessible—not merely advisable.

None of these reorientations requires abandoning the epidemiological insights Nova has generated—the evidence of harm from ultra‐processed dietary patterns is real and should motivate governance action. They require applying those insights more rigorously, more honestly, and with greater operational discipline than current Nova‐centric discourse has demonstrated.

## 7 Conclusions

As a stand‐alone public health governance framework, the primary basis for dietary recommendations to populations and regulatory guidance to governments across the full diversity of global food system contexts, Nova faces structural limitations that its most recent articulations have not resolved. Nova Group 4 conflates a nutritionally and functionally heterogeneous range of products under a single governance signal that resists meaningful product‐level differentiation. Its implicit global generalisability ignores the profound diversity of food system contexts, structural dependencies, and nutritional functions across which its recommendations would apply. And it has not generated, in over a decade of policy influence, a credible, costed, operationally grounded strategy for delivering the caloric, nutritional, and food safety functions currently performed by ultra‐processed foods to the world's food‐insecure, cold‐chain‐deficient, time‐poor and micronutrient‐deficient populations.

The appropriate response is not a categorical defence of the status quo—the epidemiological evidence of harm from the most nutritionally depleted and hyperpalatable UPF categories is too strong to dismiss—nor a retreat into dietary idealism that ignores the structural determinants of food choice for billions of people. It is the methodologically and ethically demanding work of building governance frameworks that are simultaneously evidence‐based and operationally credible: addressing demonstrable harm through proportionate, differentiated regulatory instruments; protecting the micronutrient delivery infrastructure on which the most nutritionally vulnerable populations depend; generating supply‐side investment in the food system conditions that would make structural UPF dependence progressively reducible; and placing the interests of the food‐insecure, the time‐poor, and the nutritionally vulnerable at the centre of policy design rather than at its periphery.

## Funding

The author has nothing to report.

## Disclosure

Declaration of generative AI and AI‐assisted technologies in the writing process: During the preparation of this work, the authors used Claude sonnet 4.6 (https://claude.ai) in May 2026 in order to improve the grammar and clarity of the text he drafted. After using this tool/service, the corresponding author reviewed and edited the content as needed and takes full responsibility for the content of the publication.

## Conflicts of Interest

The author declares no conflicts of interest.

## Data Availability

The author has nothing to report.
